# Dentinogenic ghost cell tumor in a pediatric patient: A case report and literature review

**DOI:** 10.1097/MD.0000000000043332

**Published:** 2025-07-18

**Authors:** Müjde Gürsu, Mehmet Altay Sevimay, Ertuğrul Çekmez, İpek Atak Seçen, Mehmet Bariş Şimşek

**Affiliations:** a Department of Oral and Maxillofacial Surgery, Gazi University Faculty of Dentistry, Ankara, Turkey; b Department of Oral Pathology, Gazi University Faculty of Dentistry, Ankara, Turkey.

**Keywords:** benign odontogenic tumor, dentinogenic ghost cell tumor, ghost cells, histopathology, pediatric patient

## Abstract

**Rationale:**

Dentinogenic ghost cell tumors (DGCTs) are rare odontogenic neoplasms constituting the solid variant of calcifying odontogenic cysts. Predominantly observed in adults, DGCTs are exceptionally rare in pediatric patients. This case report aims to contribute to the limited literature by presenting the clinical, radiographic, and histopathological findings of a pediatric patient with a DGCT and emphasizing the importance of early diagnosis.

**Patient concerns:**

A 14-year-old male presented with facial asymmetry in the left mandible.

**Diagnoses:**

Diagnostic imaging revealed a multilocular radiolucent lesion with impacted teeth, cortical expansion, and root resorption. Histopathological evaluation confirmed the DGCT diagnosis.

**Interventions:**

The lesion was treated conservatively with enucleation and curettage, followed by a 3-year follow-up.

**Outcomes:**

Regular follow-ups demonstrated progressive bone regeneration with no evidence of recurrence. No postoperative complications were observed.

**Lessons:**

Because of their high recurrence rate and potential for transformation into dentinogenic ghost cell carcinoma, early diagnosis and long-term follow-up are crucial for effective management in DGCT cases.

## 1. Introduction

Dentinogenic ghost cell tumors (DGCTs) are extremely rare benign mixed odontogenic tumors and are considered the solid, neoplastic form of calcifying odontogenic cysts (COCs).^[1]^ Over the years, ghost cell lesions have been classified in different ways. In the most recent World Health Organization (WHO) classification in 2022, COCs were classified as odontogenic cysts, their solid variants were classified as DGCTs, and their malignant form was accepted as ghost cell odontogenic carcinoma.^[2]^ Among odontogenic ghost cell lesions, DGCTs are the least common type, accounting for only 2% to 14% of all COCs.^[3]^ According to the WHO, a DGCT is characterized as a locally invasive neoplasm composed of epithelial cell islands resembling ameloblastoma in a mature connective tissue stroma. Ghost cells, representing abnormal keratinization, can be observed alongside varying amounts of dysplastic dentin.^[4]^ DGCTs typically occur between the third and fifth decades of life and are more frequently observed in males. Although they can develop in any age group, their occurrence in pediatric patients is exceptionally rare.^[5]^

The rarity of DGCTs increases the likelihood of misdiagnosis.^[6]^ Their complex histopathological features can make diagnosis challenging. Nevertheless, the presence of ghost cells, along with dentinoid and epithelial cells, allows histological differentiation from other lesions.^[7]^ Despite their benign nature, DGCTs frequently show local infiltration and a high risk of recurrence after surgical treatment, leading to the use of both conservative and radical treatment approaches. However, the importance of long-term patient follow-up is consistently emphasized to prevent potential recurrence and ensure successful outcomes. Few pediatric cases have been reported in the literature, typically supported by clinical, radiographic, and histological confirmation.^[8]^

This case report presents the management of a DGCT in the posterior mandible of a 14-year-old male patient using conservative surgical approaches instead of radical methods, which could be highly traumatic for a pediatric patient. Furthermore, this report contributes to the existing literature by emphasizing the tumor’s clinical, radiographic, and histopathological features and highlighting the high healing potential in pediatric patients.

## 2. Case presentation

Written informed consent was obtained from the patient and his legal guardian for the publication of this paper, including any associated clinical details and images.

**Figure 1.**
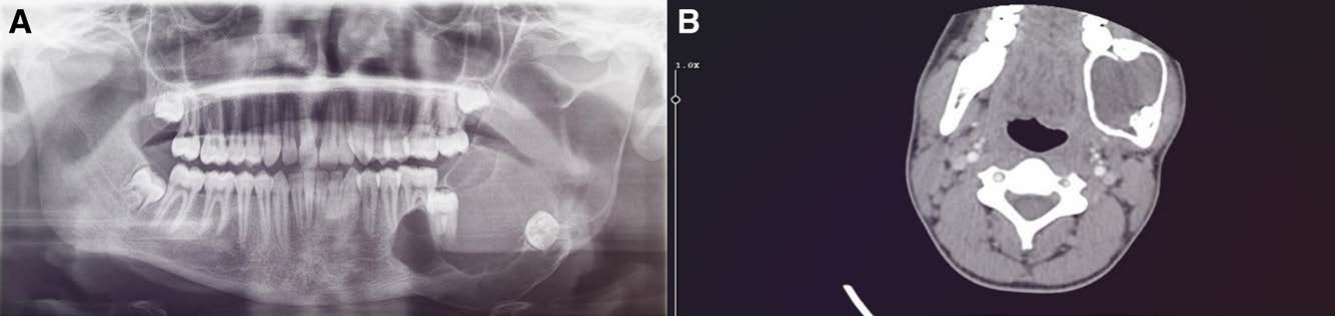
Preoperative radiological images. (A) A preoperative panoramic radiograph demonstrating a well-defined, multilocular radiolucency originating from the root apex of tooth 35, causing root resorption of tooth 36, and encompassing impacted teeth 37 and 38. (B) Preoperative axial CT image showing lesion-induced cortical expansion and the presence of impacted teeth 37 and 38. CT = computed tomography.

A 14-year-old male patient with no history of systemic diseases presented to our clinic with a 6-month history of facial asymmetry and swelling in the left mandibular region. Because he lived in a remote area with limited access to diagnostic and treatment facilities, the patient underwent prolonged antibiotic therapy to manage the swelling before seeking clinical evaluation at our clinic 6 months later. The patient reported no associated pain or history of paresthesia. Extraoral examination revealed expansion in the left posterior mandible. Intraoral examination also showed expansion in the posterior region of the mandible, with the overlying mucosa appearing normal. Vitality testing indicated that teeth 34, 35, and 36 were vital; however, mobility was detected in tooth 36. A panoramic radiograph revealed that teeth 37 and 38 were impacted, completely enclosed within a multilocular radiolucent lesion (Fig. 1A). Computed tomography imaging confirmed cortical expansion in the posterior region of the left mandible and showed that teeth 37 and 38 were impacted within the lesion (Fig. 1B).

An incisional biopsy was performed, and the solid specimen was fixed in 10% buffered formalin and analyzed by the oral pathology department. Based on the incisional biopsy, a diagnosis of DGCT was established. Following histopathological evaluation, the patient and his parents were thoroughly informed about the lesion and its high risk of recurrence. The patient was scheduled for surgery under general anesthesia via an intraoral approach.

**Figure 2.**
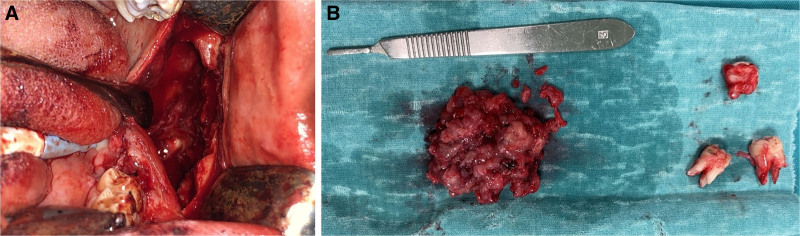
(A) Surgical enucleation and curettage of the lesion. (B) Enucleated solid tumor specimen along with extracted teeth 36, 37, and 38.

The impacted teeth 37 and 38, along with the affected and mobile tooth 36, were extracted. The solid lesion was carefully enucleated, with meticulous curettage performed to avoid damaging the surrounding neural structures (Fig. 2A). Despite the thin cortical boundary of the mandible, no pathological fracture occurred during enucleation, and mandibular continuity was preserved. The flap was then repositioned and secured with 4 to 0 resorbable sutures for primary closure.

**Figure 3.**
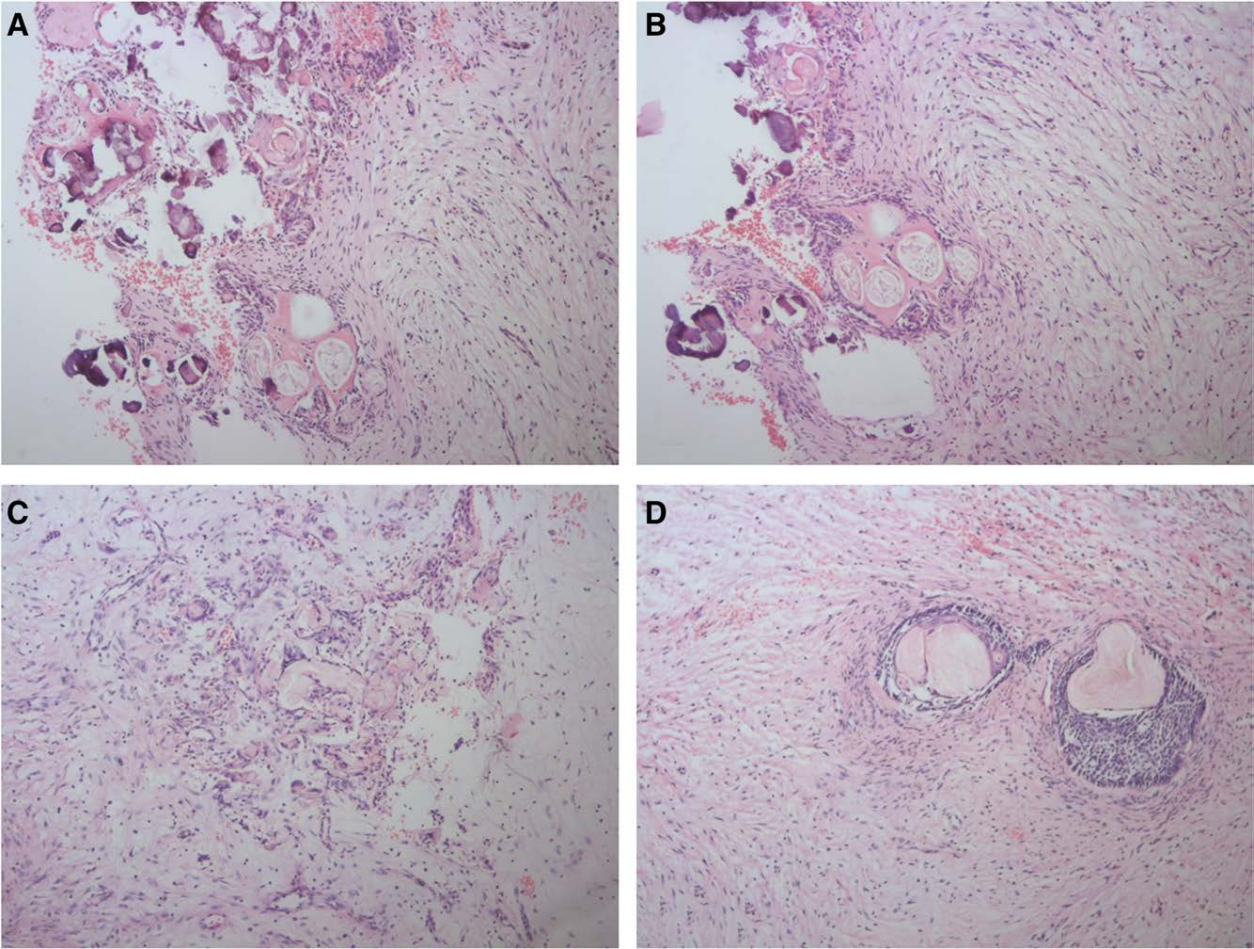
Histopathological findings. (A, B) Histological sections reveal a DGCT with a prominent cystic component. Within this cystic structure, the basal layer cells exhibit reverse polarization, and the remaining cells are composed of odontogenic epithelium (hematoxylin & eosin, 100× magnification). Pink eosinophilic ghost cells are observed in the upper layers; however, most of these cells are calcified. (C, D) Histological appearance of tumor islands located in collagenized connective tissue. In Figure D ghost cells are observed in the center of the epithelial islands showing ameloblastic features (hematoxylin & eosin, 100× magnification). DGCT = dentinogenic ghost cell tumor.

After surgery, the patient was prescribed antibiotics, analgesics, and a 0.2% chlorhexidine gluconate mouth rinse. The excised specimen was sent for histopathological examination (Fig. 2B). Histopathological examination revealed a benign odontogenic tumor with a cystic component. The tumor consisted of basal layer cells with reverse polarization and odontogenic epithelial cells containing ghost cells with dystrophic calcification (Fig. 3A, B). Loosely collagenized stroma, ghost cells within the epithelium, and epithelial islands in the stroma were also observed (Fig. 3C, D). Based on these findings, the diagnosis of DGCT was confirmed through histopathological evaluation of the excisional biopsy specimen.

**Figure 4.**
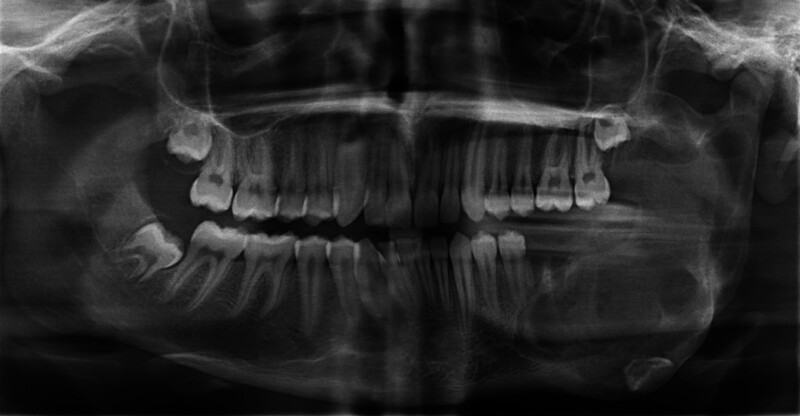
Postoperative panoramic radiograph taken on the day following surgery.

**Figure 5.**
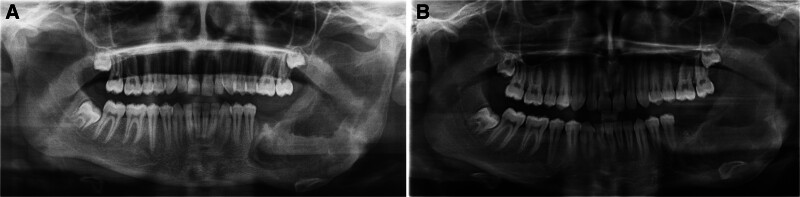
(A) Panoramic radiograph taken 1 mo after surgery. (B) Panoramic radiograph taken 3 mo after surgery.

A panoramic radiograph was taken on the day after the operation, and the patient and his parents were informed about the importance of regular follow-up (Fig. 4). The sutures were removed 10 days later, and no soft tissue dehiscence was observed. The patient exhibited no signs of postoperative infection or paresthesia. At the 1-month postoperative follow-up, teeth 34 and 35 were found to have maintained their vitality, and radiographic examination revealed no pathological findings (Fig. 5A). The 3-month postoperative radiograph showed the initiation of bone filling within the lesion cavity (Fig. 5B).

**Figure 6.**
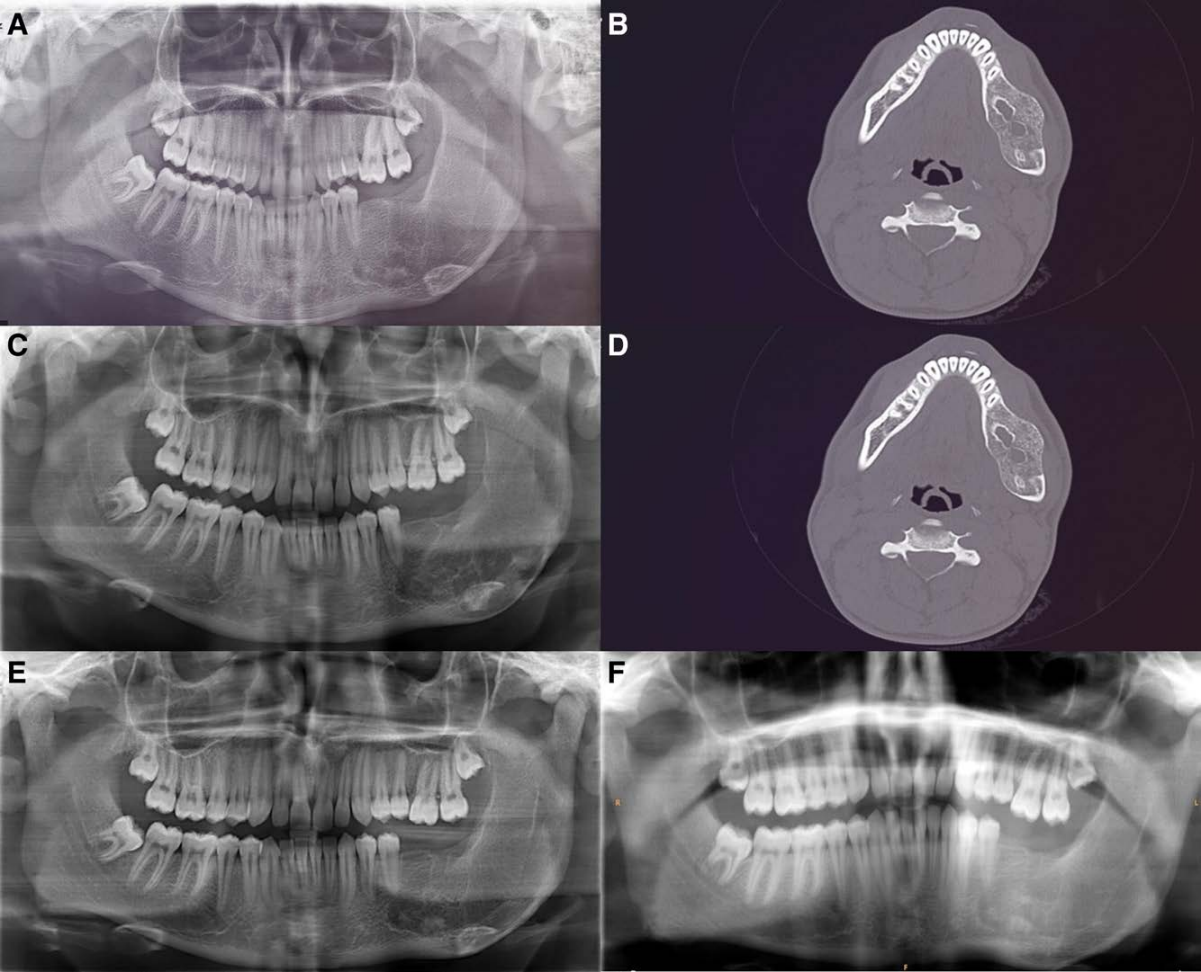
Postoperative radiographic images. (A) Panoramic radiograph 6 mo after surgery. (B) Axial cone-beam computed tomography (CBCT) view 6 mo after surgery. (C) Panoramic radiograph 12 mo after surgery. (D) Axial CBCT view 12 mo after surgery. (E) Postoperative panoramic radiograph evaluation after 2 yr. (F) Postoperative panoramic radiograph evaluation after 3 yr. CBCT = cone-beam computed tomography.

The patient maintained strict regular follow-up, and panoramic and cone-beam computed tomography images taken at the 6-month follow-up demonstrated ongoing bone healing and a reduction in expansion in the left posterior mandible (Fig. 6A, B). Panoramic and cone-beam computed tomography images taken at the 1-year follow-up revealed ongoing bone healing (Fig. 6C, D). During continued regular follow-ups, panoramic images obtained at the 2-year (Fig. 6E) and 3-year (Fig. 6F) postoperative evaluations showed bone filling and no signs of recurrence. Throughout the 3-year follow-up period, the patient exhibited no postoperative complications, such as infection, pain, or paresthesia. The patient is attending regular follow-ups to ensure long-term treatment success.

## 3. Discussion

DGCTs represent approximately 0.28% to 0.38% of all odontogenic tumors, classifying them as exceptionally rare benign odontogenic tumors with extremely infrequent occurrence in adults and children.^[6,7]^ Initially, DGCTs were considered a solid variant of COCs. However, this variant was later recognized to exhibit several key features of a solid neoplasm, leading to its reclassification as DGCTs.^[3]^ This benign neoplasm has the potential for rapid growth and, if left untreated, can reach a significant size.^[9]^ Clinically, DGCTs typically manifest as asymptomatic swelling and affect the maxilla and mandible equally, although they show a predilection for the posterior region.^[10]^ The mean age at diagnosis is 45 to 50 years, and DGCTs are slightly more common in males.^[6]^ This article adds to the limited reports of pediatric DGCTs, describing a 14-year-old male with no systemic diseases who presented to our clinic with complaints of progressively enlarging swelling and facial asymmetry in the left lower jaw for 6 months. The patient reported no other symptoms, such as pain or paresthesia, in the affected area.

DGCTs may present as intraosseous lesions (83%) or more rarely as extraosseous lesions (17%) originating from the gingiva or alveolar mucosa. Both intraosseous and extraosseous DGCT variants share comparable histopathological characteristics.^[6]^ The size of an intraosseous DGCT ranges from 1 cm to over 10 cm in diameter, and they are typically asymptomatic. Clinical manifestations of intraosseous DGCTs include cortical expansion and clinically visible swelling. These lesions are often detected incidentally during routine radiographic examinations and may be accompanied by dental mobility.^[11]^ Unlike intraosseous lesions, extraosseous DGCT lesions are more common in older individuals and tend to localize in anterior, edentulous regions.^[12]^ In the present case, clinical examination of the patient revealed expansion in the left mandibular posterior area and mobility of tooth 36. Apart from these findings, no other pathological signs were observed during the intraoral examination.

Radiographically, DGCTs typically present as well-defined unilocular or multilocular lesions, which may appear radiolucent, radiopaque, or mixed, depending on the amount of calcification within the lesion.^[3]^ Root resorption, displacement of adjacent teeth, and the presence of impacted teeth can also occur.^[11]^ In the present case, panoramic radiography revealed a well-defined multilocular radiolucent lesion. The lesion completely enveloped impacted teeth 37 and 38, causing the displacement of impacted tooth 38 and the mandibular canal. Additionally, root resorption of tooth 36 was observed. The well-defined multilocular radiolucent lesion, along with findings of tooth displacement and root resorption, were consistent with previously reported cases. Computed tomography imaging of the presented case further demonstrated a well-defined multilocular radiolucent area with cortical bone expansion, findings that align with previous reports. To establish a definitive diagnosis, an incisional biopsy followed by histopathological examination was performed.

When unilocular or multilocular radiolucent or mixed radiographic images are observed in the jawbones, a broad spectrum of odontogenic and non-odontogenic benign and malignant lesions should be considered in the differential diagnosis. For the differential diagnosis of DGCTs, lesions such as adenomatoid odontogenic tumors, calcifying epithelial odontogenic tumors, odontogenic myxoma, and ameloblastoma should be considered. However, the identification of ghost cells, dysplastic dentin, and other calcifications often helps differentiate DGCTs from these lesions.^[13]^ Histopathological evaluation remains the only reliable method to confirm DGCT diagnosis as it exhibits nonspecific clinical and radiographic characteristics.

Histopathologically, DGCTs are benign odontogenic tumors composed of neoplastic epithelial islands resembling ameloblastoma, along with ghost cells and dentin.^[7]^ As outlined by the WHO, the identification of ghost cells and dentin exceeding 1% to 2% is regarded as a key diagnostic feature of DGCTs.^[6]^

The most characteristic histopathological feature of DGCTs is the presence of ghost cells within tumor islands, which are sparsely distributed in the connective tissue. Ghost cells are defined as cell clusters characterized by the absence of a nucleus, eosinophilic cytoplasm, and inherent resistance to degradation. The cellular outline of ghost cells is typically discernible; however, under microscopic examination, groups of ghost cells frequently exhibit a fused appearance.^[14]^ Occasionally, dystrophic calcification may occur within ghost cells, appearing as fine basophilic granules or small spherical bodies. In certain instances, particularly in cases of DGCTs, ghost cells from tumor islands may interact with the connective tissue. This interaction can trigger a foreign body reaction characterized by the formation of multinucleated giant cells.^[15]^ In the present case, dystrophic calcification was observed in most ghost cells. The formation of ghost cells is a topic of extensive debate. Most authors regard ghost cells as representing either abnormal or incomplete keratinization, or even true keratinization.^[16]^ Although some researchers have proposed that this keratinization results from squamous cell degeneration, others have hypothesized that it is caused by ischemia-induced calcification following squamous metaplasia. Another theory suggests that ghost cells originate from the metaplastic transition of odontogenic epithelium and contain both normal and abnormal keratin.^[14]^ Additionally, some researchers have speculated that ghost cells may result from defective enamel matrix formation in the odontogenic epithelium.^[14,15]^ The prevailing hypothesis is that ghost cells form because of abnormal keratogenesis. This theory is supported by the observation that ghost cells do not stain with normal cytokeratin markers.^[17]^ Advances in molecular technologies have revealed that tumors in the family of ghost cell lesions are associated with beta-catenin missense mutations in the WNT signaling pathway.^[16,17]^ Mutations in the *CTNNB1* gene, which encodes beta-catenin, have been implicated in the development of these tumors.^[18]^ This finding supports the notion that ghost cells exhibit keratin-like properties, although the keratin in ghost cells is considered abnormal. This abnormality may explain the propensity of ghost cells to accumulate dystrophic calcifications and induce a foreign body reaction when in contact with connective tissue. In the presented case, histopathological examination revealed odontogenic epithelial cells with basal layer cells exhibiting reverse polarization, along with ghost cell populations and dystrophic calcifications. Additionally, the tumor stroma was observed to be loosely collagenized, with ghost cells not only present within the epithelium but also forming clusters in the stroma. According to this histopathological evaluation, the case was diagnosed as a DGCT.

Early diagnosis of DGCTs is critical for improving patient prognosis and establishing an appropriate treatment plan. Intraosseous lesions are typically managed with surgical methods, such as enucleation, segmental resection, or partial resection. Long-term follow-up is recommended, as recurrence has been reported 1 to 5 years after segmental or partial resection, as well as after enucleation. Intraosseous DGCTs are aggressive neoplasms with infiltrative behavior, exhibiting recurrence rates as high as 71%.^[19]^ The infiltrative growth pattern observed in the intraosseous form of DGCTs may explain their high recurrence rates.^[8]^ A study by Sun et al described 7 patients with intraosseous DGCTs; 5 of them underwent conservative treatment and experienced recurrence, whereas no recurrence was observed in the 2 patients who received aggressive surgical resection.^[19]^ In contrast to intraosseous DGCTs, peripheral DGCTs exhibit less aggressive behavior and are generally considered less prone to recurrence.^[7]^ Consequently, extraosseous lesions are typically treated with local excision.^[12]^ Additionally, malignant transformation of DGCTs into odontogenic ghost cell carcinoma has been reported, although such cases are extremely rare.^[20]^ Given the high recurrence rates and the possibility of progression to the malignant form, known as ghost cell odontogenic carcinoma, long-term follow-up is strongly recommended.^[4]^ In the present case, after histopathological evaluation, treatment options were thoroughly explained to the patient and his parents, emphasizing the high risk of recurrence. Radical treatment options such as resection, which are typically recommended for intraosseous DGCT lesions due to their recurrence risk, were deemed excessively traumatic for this pediatric patient. Considering pediatric patients’ high healing potential, enucleation and curettage were chosen as the treatment. The patient has been monitored regularly at 3-month intervals following enucleation and curettage, with no recurrence observed during the 3-year follow-up period. Furthermore, the patient’s facial asymmetry has resolved, and no symptoms have been reported.

Several similar cases in the literature support the effectiveness of conservative treatment in pediatric patients. Agrawal et al reported a case of DGCT in a 14-year-old male presenting with a 4-month history of swelling in the lower right premolar–molar region. Radiographic evaluation revealed a well-defined, multilocular mixed lesion with cortical expansion, root resorption, and displacement of unerupted teeth. The lesion was surgically enucleated, and the patient was placed under regular follow-up. As reported, no recurrence has been observed to date, and the patient remains asymptomatic.^[12]^ Ravi et al reported a case of DGCT in a 12-year-old girl presenting with swelling in the anterior mandible crossing the midline. Radiographic examination revealed a well-defined mixed radiolucent–radiopaque lesion with displacement of adjacent teeth. The lesion was treated by local excision, and complete healing was observed with no recurrence during a 2-year follow-up.^[21]^ Another report by Urs et al involved a 17-year-old male with a mandibular DGCT associated with a composite odontoma. The lesion exhibited multilocular radiolucency with surrounding radiopaque components. The authors performed enucleation and peripheral ostectomy, and the patient remained recurrence-free at 18 months.^[22]^ In contrast, Juneja et al reported a case involving a 14-year-old female patient who presented with a long-standing swelling in the right posterior mandible, accompanied by intermittent pain, pus discharge, difficulty in mastication, and reduced mouth opening. Clinical and radiographic examination revealed a well-defined mixed radiolucent–radiopaque lesion causing cortical expansion and displacement of adjacent teeth. The lesion was treated with segmental resection of the mandible. No recurrence was observed during the first 6 months following treatment.^[23]^

The present case report contributes to the limited literature by highlighting the occurrence of DGCTs in pediatric patients. By emphasizing the clinical, radiographic, and histopathological features, this report enhances the understanding of this rare lesion and supports accurate diagnosis and treatment in younger patients.

## 4. Conclusions

DGCTs are extremely rare odontogenic neoplasms, with very few cases reported in the pediatric population. Considering their high recurrence potential, long-term follow-up is essential following surgical treatment. In pediatric patients with high healing potential, there is a need for further studies incorporating long-term follow-up to better understand postsurgical outcomes.

## Acknowledgments

This article is a revised and expanded version of a paper titled “Dentinogenic Ghost Cell Tumor Associated with Impacted Teeth in a Pediatric Patient: A Case Report.” which was originally presented as a poster presentation at the Turkish Association of Oral and Maxillofacial Surgery 30th International Scientific Congress, held in Antalya, Turkey, from November 17 to 21, 2023.

## Author contributions

**Conceptualization:** Müjde Gürsu, Mehmet Altay Sevimay, Ertuğrul Çekmez, Mehmet Bariş Şimşek.

**Data curation:** Müjde Gürsu, Mehmet Altay Sevimay, Ertuğrul Çekmez, İpek Atak Seçen.

**Investigation:** Müjde Gürsu, Mehmet Altay Sevimay, İpek Atak Seçen.

**Resources:** Müjde Gürsu, Ertuğrul Çekmez, İpek Atak Seçen.

**Supervision:** Mehmet Bariş Şimşek.

**Validation:** Mehmet Bariş Şimşek.

**Visualization:** Müjde Gürsu, Mehmet Altay Sevimay, İpek Atak Seçen.

**Writing – original draft:** Müjde Gürsu, Mehmet Altay Sevimay, İpek Atak Seçen.

**Writing – review & editing:** Müjde Gürsu, Mehmet Altay Sevimay.
